# Selecting high-quality negative samples for effectively predicting protein-RNA interactions

**DOI:** 10.1186/s12918-017-0390-8

**Published:** 2017-03-14

**Authors:** Zhanzhan Cheng, Kai Huang, Yang Wang, Hui Liu, Jihong Guan, Shuigeng Zhou

**Affiliations:** 1 School of Computer Science, Fudan University, Handan Road, Shanghai, 200433 China; 2 The Bioinformatics Lab at Changzhou NO. 7 People’s Hospital, Changzhou, Jiangsu, 213011 China; 3 Department of Computer Science and Technology, Tongji University, Shanghai, 201804 China; 4 Lab of Information Management, Changzhou University, Changzhou, 213164 China; 5 School of Computer Science, Jiangxi Normal University, Nanchang, 330022 China

**Keywords:** Protein-RNA interactions, Reliable negative samples, Unreliable negative samples

## Abstract

**Background:**

The identification of Protein-RNA Interactions (PRIs) is important to understanding cell activities. Recently, several machine learning-based methods have been developed for identifying PRIs. However, the performance of these methods is unsatisfactory. One major reason is that they usually use unreliable negative samples in the training process.

**Methods:**

For boosting the performance of PRI prediction, we propose a novel method to generate reliable negative samples. Concretely, we firstly collect the known PRIs as positive samples for generating positive sets. For each positive set, we construct two corresponding negative sets, one is by our method and the other by random method. Each positive set is combined with a negative set to form a dataset for model training and performance evaluation. Consequently, we get 18 datasets of different species and different ratios of negative samples to positive samples.

Secondly, sequence-based features are extracted to represent each of PRIs and protein-RNA pairs in the datasets. A filter-based method is employed to cut down the dimensionality of feature vectors for reducing computational cost. Finally, the performance of support vector machine (SVM), random forest (RF) and naive Bayes (NB) is evaluated on the generated 18 datasets.

**Results:**

Extensive experiments show that comparing to using randomly-generated negative samples, all classifiers achieve substantial performance improvement by using negative samples selected by our method. The improvements on accuracy and geometric mean for the SVM classifier, the RF classifier and the NB classifier are as high as 204.5 and 68.7%, 174.5 and 53.9%, 80.9 and 54.3%, respectively.

**Conclusion:**

Our method is useful to the identification of PRIs.

## Background

Exploring the interactions between proteins and RNAs can help us to understand the mechanisms of life, such as the protein translation process [1–3], gene expression [4, 5], RNA post-transcriptional modification [6–8], cellular regulation [9, 10].

A lot of effort has been put on the identification of PRIs using traditional experimental methods and post-experimental methods. As experimental methods consume more time and money than post-experimental methods, the latter is gaining more and more attention. There are mainly two categories of post-experimental methods: 1)structural & chemical-based methods and 2)computational methods.

The first category of methods attempted to analyze the interacting mechanism of protein and RNA at structural and chemical levels. For example, Jones et al. [11] focused on analyzing protein-RNA complexes, and obtained the physical-chemical properties of RNA-binding residues and the distribution of atom-atom within the complexes. With protein-RNA experimental data, Ellis et al. [12] presented a statistics on properties of binding residues bounding to functional various RNAs. Besides, some function-based works [13, 14] also discussed the protein-RNA interactions.

As for computation-based methods, several machine learning techniques have been employed on identifying PRIs, such as random forest (RF), Naive Bayes (NB) and support vector machine (SVM). Pancaldi et al. [15] used RF and SVM for identifying PRIs by considering more than 100 properties of RNAs and proteins. Instead, Muppirala et al. [16] used only protein and RNA sequence information for predicting interactions. Similarly, Wang et al. [17] improved the Naive Bayes (ENB) classifiers for predicting PRIs with only sequence data. Recently, we also proposed learning method [18] with only positive and unlabeled samples on PRIs prediction.

Compared with structural & chemical-based methods, computational methods are more efficient and effective. However, the performance of computational methods heavily depends on the quality of training datasets, which usually consist of positive samples and negative samples. Here, positive samples are not the problem. The difficulty lies in that we do not have experimentally-validated negative samples. Current works [16, 17] addressed this problem by randomly pairing RNAs and proteins and then removing these pairs included in the positive set. In this paper, we call this method *random method* or *traditional method*. Obviously, random negative samples must not be real negative samples. So the quality of random negative sets cannot be guaranteed. This will unavoidably impact prediction performance of classifiers trained on datasets with random negative samples.

This paper addresses how to select highly reliable negative samples to improve PRI prediction. To this end, we present an effective method *FIRE* — the abbreviation of ***FI***nding ***R***eliable n***E***gative samples). The basic idea of our method is like this: given a known PRI of protein *i* and RNA *j*, for a protein *k*, the more difference between protein *i* and protein *k*, the less possibility that protein *k* interacts RNA *j*.

We first construct positive sets using known PRIs. Given a positive set, we establish two negative sets: one is by random method and the other by our method. And the positive set is combined with each of the two negative sets to form a dataset for model training and performance evaluation. In such a way, we construct 18 datasets of different species and different ratios of negative samples to positive samples. Then, we extract the features of each pair of protein and RNA. Here, each feature is composed of a conjoint triad of vicinal amino acids and a *k* nucleotide acids. To cutoff computational cost, a filter-based feature selection method is employed to reduce the dimensionality of feature vectors. Finally, we conduct extensive experiments to evaluate the proposed method by training and testing SVM, RF and NB classifier on the 18 datasets. The experimental results show that these classifiers perform much better using the negative samples generated by our method than using random negative samples.

## Methods

**Fig. 1:**
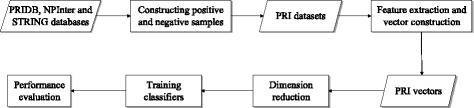
The framework of this work. Here, *rectangles* are executive modules, and *parallelograms* are data modules

We collected non-redundant known PRIs as positive samples, and generated 18 datasets based on our method and the random method, which were used to evaluate the performance of PRI prediction by SVM, RF and NB classifiers. Figure 1 is the procedure of our method, which contains five steps: 1) Generating negative datasets by using our method *FIRE* and the random method; 2) Constructing feature vectors for each pair of protein-RNA; 3) Reducing the dimension of feature vectors; 4) Training classifiers; 5) Performance evaluation.

### Datasets

We constructed 9 non-redundant positive PRI sets from PRIDB [19], NPInter [20], 9 reliable negative sets based on the positive sets and the STRING [21] database by our method, and 9 random negative sets with the random method. The procedure for negative sample construction will be detailed later. Each positive set is merged with a negative set to construct a PRI dataset, consequently 18 PRI datasets in total are constructed. PRIDB is a database of protein-RNA interfaces calculated from protein-RNA complexes in PDB [22]. NPInter is a complete database covering eight-category functional interactions between proteins and noncoding RNAs of six model organisms, including *Caenorhabditis elegans*, *Drosophila melanogaster*, *Escherichia coli*, *Homo sapiens*, *Mus musculus* and *Saccharomyces cerevisiae*. STRING is an updated online database resource Search Tool for the Retrieval of Interacting Genes, it provides uniquely comprehensive coverage and ease of access to both experimental and predicted protein-protein interaction (PPI) information.

The 18 datasets are divided to 3 groups. The first group of datasets (denoted group 1) contain 336 experimental-validated PRIs that are used as positive samples, which are related to the six organisms above and constructed from the NPInter and STRING databases. This group consists of six sub-datasets (named by SO) as follows:

1. The first sub-dataset (*SO*_*reliable*_1:1_) contains 168 positive samples and 168 reliable negative samples generated by our method, the ratio of positives to negatives is 1:1;
2. The second sub-dataset (*SO*_*reliable*_2:1_) contains 336 positive samples and 168 reliable negative samples, the ratio is 2:1;
3. The third sub-dataset (*SO*_*reliable*_1:2_) contains 168 positive samples and 336 reliable negative samples, the ratio is 1:2;
4. The fourth sub-dataset (*SO*_*random*_1:1_) contains 168 positive samples and 168 random negative samples generated by the random method, and the ratio of positives to negatives is 1:1;
5. The fifth sub-dataset (*SO*_*random*_2:1_) contains 336 positive samples and 168 random negative samples, the ratio is 2:1;
6. The last sub-dataset (*SO*_*random*_1:2_) contains 168 positive samples and 336 random negative samples, the ratio is 1:2.

The second group of datasets (denoted as group 2) includes 1320 experimental-validated homo species PRIs used as positive samples, which are extracted from the PRIDB and STRING databases, it also consists of six sub-datasets. Following the nomenclature of the first group of datasets, these PRI datasets are named as *HOMO*_*reliable*_1:1_, *HOMO*_*reliable*_2:1_, *H* *O* *M* *O* _*reliable*_1:2_, *HOMO*_*random*_1:1_, *HOMO*_*random*_2:1_,*HOMO*_*random*_1:2_.

The third group of datasets (denoted as group 3) has 114 experimental-validated mouse PRIs as positive samples, which also consists of six sub-datasets: *MUS*_*reliable*_1:1_, *MUS*_*reliable*_2:1_, *MUS*_*reliable*_1:2_, *MUS*_*random*_1:1_, *MUS*_*random*_2:1_, *MUS*_*random*_1:2_.

Table 1 gives the statistics of the total 18 PRI datasets.

**Table 1:** The 18 PRI datatsets used in this paper

Datesets	# Positive samples	# Negative samples
*SO*_*reliable*_1:1_	168	168
*SO*_*reliable*_2:1_	336	168
*SO*_*reliable*_1:2_	168	336
*SO*_*random*_1:1_	168	168
*SO*_*random*_2:1_	336	168
*SO*_*random*_1:2_	168	336
*HOMO*_*reliable*_1:1_	660	660
*HOMO*_*reliable*_2:1_	1320	660
*HOMO*_*reliable*_1:2_	660	1320
*HOMO*_*random*_1:1_	660	660
*HOMO*_*random*_2:1_	1320	660
*HOMO*_*random*_1:2_	660	1320
*MUS*_*reliable*_1:1_	57	57
*MUS*_*reliable*_2:1_	114	57
*MUS*_*reliable*_1:2_	57	114
*MUS*_*random*_1:1_	57	57
*MUS*_*random*_2:1_	114	57
*MUS*_*random*_1:2_	57	114

#### Construction of random negative samples

**Fig. 2:**
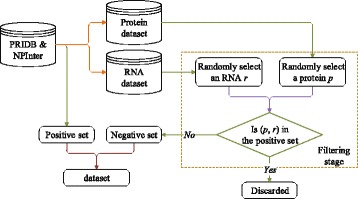
The flowchart of constructing random negative samples

Previous works [16, 17] randomly select negative samples, the underlying hypothesis is: if there is no validated interaction between a protein and a RNA, then the protein and the RNA constitute a negative sample. Obviously, the hypothesis is not completely reasonable. The flowchart for generating random negative samples is shown in Fig. 2.

In Fig. 2, the major steps of the random method are as follows:

1. Each PRI extracted from PRIDB and NPInter is included in the positive set. From the positive set, we can get a set *P* of proteins and a set *R* of RNAs, each protein/RNA in *P*/*R* is involved in at least a positive PRI.
2. For each protein *p* in *P* and each RNA *r* in *R*, there is a corresponding protein-RNA pair (*p*, *r*).
3. If (*p*, *r*) is not included in the positive set, it is a negative sample.
4. The positives and negatives are merged to a PRI dataset.

#### Construction of reliable negative samples

**Fig. 3:**
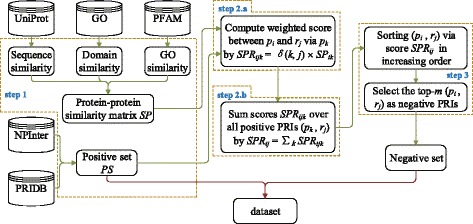
The flowchart of constructing reliable negative samples

The basic idea of our method is like this: for an experimentally-validated PRI of protein *p* and RNA *r*, *r* is highly possible to interact with any protein *p* ^′^ similar to *p*. On the contrary, if protein *p* ^′^ is dissimilar to *p*, there is low possibility that *p* ^′^ interacts *r*. Based on this idea, we propose the method *FIRE* to construct reliable negative PRIs. The flowchart of FIRE is shown in Fig. 3. Concretely, for each positive PRI (*p*, *r*), we try to find any protein *p* ^′^ that is as much dissimilar as possible to *p*. If (*p* ^′^, *r*) is not an experimentally-validated PRI, then it is selected as a negative PRI.

We first compute the similarity between each pair of proteins based on three different data sources, then we combine these similarity scores as a final score to measure the similarity between the two proteins. Detail is delayed to “Protein-protein similarity computation” section.

The procedure of our method FIRE is as follows:

1. Construct the positive set *PS* of PRIs based on the PRIDB and NPInter databases, and compute the similarity matrix *SP* of proteins involved in *PS* as in “Protein-protein similarity computation” section.
2. For protein *p*_*i*_ and RNA *r*_*j*_ that do not form a positive PRI in *PS*, i.e., (*p*_*i*_, *r*_*j*_) ∉*PS*, compute a score between *p*_*i*_ and *r*_*j*_ as follows:
  - If protein *p*_*k*_ (*k*≠*i*) and *r*_*j*_ forms a PRI in the positive PRI set *PS*, then the score *SPR*_*ijk*_ indicating the confidence of (*p*_*i*_, *r*_*j*_) being a positive PRI via protein *p*_*k*_ can be evaluated via *SP*_*ik*_, which is the similarity between *p*_*i*_ and *p*_*k*_.
  - As there may be multiple (say *n*) positive PRIs involving *r*_*j*_ in *PS*, we aggregate the scores *SPR*_*ijk*_ over all positive PRIs (*p*_*k*_, *r*_*j*_) (*k*≠*i* and *k*=1..*n*) as follows:
    1 $$\begin{array}{*{20}l}  SPR_{ij} = \sum\limits_{k=1}^{n}{SPR_{ijk}}=\sum\limits_{k=1}^{n}{\delta(k,j)\times SP_{ik}}, \end{array} $$
*SPR*_*ij*_ indicates the confidence of (*p*_*i*_, *r*_*j*_) being a positive PRI, *δ*(*i*,*j*)=1 if (*p*_*k*_, *r*_*j*_) is a positive PRI, otherwise 0.
3. As (*p*_*i*_, *r*_*j*_) ∉*PS*, it is a potential negative PRI. Sorting all generated potential PRIs (*p*_*i*_, *r*_*j*_) via their scores *SPR*_*ij*_ in increasing order, the top-*m* protein-RNA pairs in the sorted list are taken as negative PRIs if *m* negative PRIs are to be generated.

#### Protein-protein similarity computation

We compute the similarity between any two proteins involved in the positive set based on three types of data sources: sequence information, functional annotations and protein domains, these computed similarities are called *sequence similarity*, *functional annotation semantic similarity* and *protein domain similarity*, which are merged to get the final similarity of the two proteins.

*Sequence similarity (SS)*. Protein sequences are obtained from the UniProt database [23]. We compute sequence similarity between two proteins using a normalized version of Smith-Waterman score [24]. The normalized Smith-Waterman score between two proteins *p*_*i*_ and *p*_*j*_ is *nsw*(*p*_*i*_,*p*_*j*_)=$sw(p_{i}, p_{j})/\sqrt {sw(p_{i}, p_{j})}\sqrt {sw(p_{j}, p_{j})}$ where *sw*(.,.) means the original Smith-Waterman score. By applying this operation to protein pair *p*_*i*_ and *p*_*j*_, we can obtain their sequence similarity *SS*(*p*_*i*_,*p*_*j*_)=(*nsw*(*p*_*i*_,*p*_*j*_) + *nsw*(*p*_*j*_,*p*_*i*_))/2.

*Functional annotation semantic similarity (FS)*. GO annotations are downloaded from the GO database [25]. Semantic similarity between each pair of proteins is calculated based on the overlap of the GO terms associated with the two proteins [26]. All three types of GO are used in the computation as similar RNAs are expected to interact with proteins that act in similar biological processes, or have similar molecular functions or reside in similar cell compartments. We compute the Jaccard value [27] with respect to the GO terms of each pair of proteins as their similarity. The Jaccard score between term sets *t*_*i*_ and *t*_*j*_ of proteins *p*_*i*_ and *p*_*j*_ is defined as |*t*_*i*_∩*t*_*j*_|/|*t*_*i*_∪*t*_*j*_|, which is the ratio of the number of common terms between proteins *p*_*i*_ and *p*_*j*_ to the total number of terms of *p*_*i*_ and *p*_*j*_, which is used as the functional annotation semantic similarity *FS*(*p*_*i*_,*p*_*j*_) of proteins *p*_*i*_ and *p*_*j*_.

*Protein domain similarity (DS)*. Protein domains are extracted from Pfam database [28]. Each protein is represented by a domain fingerprint (binary vector) whose elements encode the presence or absence of each retained Pfam domain by 1 or 0, respectively. We compute the Jaccard value of any two proteins *p*_*i*_ and *p*_*j*_ with their domain fingerprints as their similarity *DS*(*p*_*i*_,*p*_*j*_).

For proteins *p*_*i*_ and *p*_*j*_, we compute the aggregated similarity (AS) by merging the three different similarity measures above as follows:

2 $$ AS(p_{i}, p_{j}) = (SS(p_{i}, p_{j})+FS(p_{i}, p_{j})+DS(p_{i}, p_{j}))/3.  $$

### PRI feature vectors

Existing works [29–31] found that properties of amino acids are effective in protein classification. To reduce the dimensionality of protein representation, Shen et al. [32] classified the 20 amino acid residues as seven classes according to their physicochemical properties, meanwhile the concept of conjoint triads were also proposed to represent the protein properties. Wang et al. [17] further reduced the dimension of feature vector by encoding the 20 amino acids residues into four classes: {*DE*}, {*HRK*}, {*CGNQSTY*}, and {*AFILMPVW*}. In this work, we use the same strategy for encoding protein sequences.

#### Feature construction

To compute protein feature vectors, we used conjoint triads as protein properties as in [16, 17, 32]. 3 continuous amino acids constitute a conjoint triad, we can get 64 (4×4×4) classes of conjoin triads. Note that two triads are treated as the same class if their residues in the corresponding positions belong to the same class. For RNA sequences, we used *k*-nucleotide acids (*k*-NAs) as properties. A *k*-NAs refers to a unit of *k* continuous nucleotide acids. *k*-NAs of size 1 (i.e. *k*=1) are called “uniNAs”, size 2 (i.e. *k*=2) are called “biNAs”, size 3 (i.e. *k*=3) are called “triNAs”, size 4 or more (i.e. $k\geqslant 4$) are simply called “*k*-NAs”. Because RNA sequences contain only the four bases A, U, C, G, we have 4 unique uniNAs, 4^2^ unique biNAs and 4^3^ unique triNAs. Finally, by pairing the *k*-NAs (*k*=1,2,3) and triads, we can get at most 256 (64×4) 4-mers, 1024 (64×4^2^) 5-mers and 4096 (64×4^3^) 6-mers, each of which is composed of a conjoint triad and a uniNA, biNA and triNA respectively. In the sequel, we also call 4-mers, 5-mers, 6-mers as type 1, 2, 3 (*k*+3)-mers.

Table 2 gives the combination of triads and *k*-NAs examples. For a pair of hypothetical amino acid sequence *DPPVPPPPPV* and nucleotide acids sequence *CCUCU*, two classes of triads {DPP}, {PPV, PVP, VPP, PPP} (note that ‘P’ and ‘V’ belong to the same class), three classes of 3-NAs {CCU}, {CUC} and {UCU}, three classes of 2-NAs {CC}, {CU} and {UC} and two classes of 1-NAs {C} and {U} are generated. Hence, we can get the following 15 6-mers by matching the 3-NAs and triads: CCU-DPP, CCU-PPV, CCU-PVP, CCU-VPP, CCU-PPP, CUC-DPP, CUC-PPV, CUC-PVP, CUC-VPP, CUC-PPP, UCU-DPP, UCU-PPV, UCU-PVP, UCU-VPP and UCU-PPP, and 15 5-mers by matching the 2-NAs and triads: CC-DPP, CC-PPV, CC-PVP, CC-VPP, CC-PPP, CU-DPP, CU-PPV, CU-PVP, CU-VPP, CU-PPP, UC-DPP, UC-PPV, UC-PVP, UC-VPP and UC-PPP, and 10 4-mers by matching the 1-NAs and triads: C-DPP, C-PPV, C-PVP, C-VPP, C-PPP, U-DPP, U-PPV, U-PVP, U-VPP, U-PPP.

**Table 2:** An example of feature extraction for a pair of protein and RNA sequences

Protein sequence	*D* *P* *P* *V* *P* *P* *P* *P* *P* *V*
RNA sequence	*C* *C* *U* *C* *U*
Triads	{*DPP*}, {*PPV*,*PVP*,*VPP*,*PPP*}
3-NAs	{*CCU*}, {*CUC*}, {*UCU*}
2-NAs	{*CC*}, {*CU*}, {*UC*}
1-NAs	{*C*}, {*U*}

#### Feature value computation

In order to discriminate the significance of different types of features in a feature vector, we introduce the concept of concentration of different features. Denote the number of unique (*k*+3)-mers of type *i* as *N*_*i*_. The concentration of type *i* is the ratio of *N*_*i*_ to the total number of unique (*k*+3)-mers, that is,

3 $$ C_{i}= \frac{N_{i}}{\sum_{j=1}^{3}{N_{j}}},\qquad i = 1, 2, 3.  $$

For example, the number of unique 6-mers is 64×4^3^. The total number of unique (*k*+3)-mers used in this study is 5376, therefore the concentration of 6-mers is *C*_3_=4096/5376=0.762. Then, the elements of a feature vector are calculated by

4 $$ f_{j}= t_{j}\times C_{i},\qquad 1\leq j \leq 5376  $$

Above, *t*_*j*_ is the occurrence frequency of a certain unique (*k*+3)-mer of type *i*. A feature vector contains 5376 dimensions, each of which corresponds to a unique (*k*+3)-mer of a certain type *i* (*i*=1, 2 and 3). Within a vector, the dimensions are arranged in the order of 6-mers, 5-mers and 4-mers. Then *f*_*i*_ is further normalized to *ff*_*i*_ as follows:

5 $$ ff_{j}= \frac{f_{j}-f_{min}}{f_{max}-f_{min}}  $$

where *f*_*max*_ and *f*_*min*_ denote the maximum and the minimum of all *f*_*j*_ (*j*=1,2,…,5376), respectively.

#### Feature reduction

In order to reduce the computational cost, we employed a filter-based method for cutting down the dimension of feature vectors.

For the *i*-th feature *ff*_*j*_(*i*) of the *j-th* vector, let *F*(*i*)_*p*_ and *F*(*i*)_*n*_ denote its occurrence frequency in the positive and negative sample set respectively, which are calculated by

6 $$\begin{array}{*{20}l} F(i)_{p}=\sum\limits_{j=1}^{N}{ff_{j}(i)},~vector~j~\in~the~positive~set, \end{array} $$

7 $$\begin{array}{*{20}l} F(i)_{n}=\sum\limits_{j=1}^{M}{ff_{j}(i)},~vector~j~\in~the~negative~set, \end{array} $$

where *N* and *M* are the numbers of positives and negatives in the dataset.

*F*(*i*)_*p*_ and *F*(*i*)_*n*_ are further normalized to *FF*(*i*)_*p*_ and *FF*(*i*)_*n*_ as in Eq. (5), and then the final score of each feature is defined as follows:

8 $$ FScore(i)= \frac{FF(i)_{p}}{FF(i)_{n}},\qquad i = {1, 2, \ldots, 5376}.  $$

Our objective is to choose those discriminative features that either frequently occur in the positive set but seldom occur in the negative set, or frequently occur in the negative set but rarely occur in the positive set. In such a way, we choose the features that help us to distinguish positive samples from negative samples.

As *FScore*(*i*) measures the relative enrichment of the *i*-th feature in the positives over the negatives, it can be regarded as an indicator of the usefulness of the *i*-th feature. Based on the calculated *FScore* values, the most “useful” features that have the largest or smallest *FScore* values are selected to represent the PRI pairs. Suppose that we reduce the PRI vectors to *k* dimensions, we select the $\frac {k}{2}$ features with the largest *FScore* values and the $\frac {k}{2}$ features with the smallest *FScore* values to represent the *k*-dimension PRI vectors. In our work, *k* is set to 1000.

### The classifiers and performance metrics

As several studies have successfully used random forest (RF), naive Bayes (NB) and support vector machine (SVM) to predict PRIs [15–17], we also use them to evaluate our method by 10-fold cross validation.

Four widely-used performance metrics, *sensitivity* (SE), *specificity* (SP), *accuracy* (ACC) and *geometric mean* (GM) are used in this paper. GM is commonly used for class-imbalance learning [33] because it can give a more accurate evaluation on imbalanced data. Therefore, for the imbalance datasets, we pay more attention to GM rather than ACC. These metrics are evaluated as follows:

9 $$ SE = \frac{TP}{TP+FN},  $$

10 $$ SP = \frac{TN}{TN+FP},  $$

11 $$ GM = \sqrt{SE\times SP},  $$

12 $$ ACC = \frac{TP+TN}{TP+FN+TN+FP},  $$

where *TP* is the number of true positives, *TN* is the number of true negatives, *FP* is the number of false positives, and *FN* is the number of false negatives.

In addition, we also use AUC (Area Under the receiver operating characteristic (ROC) Curve) to evaluate prediction performance in some experiments. AUC falls between 0 and 1. The maximum value 1 means a perfect prediction. For a random guess, the value of AUC is close to 0.5.

## Results and Discussion

In our experiments, eighteen PRI datasets are used, these datasets either contain PRI data of different species or have different ratios of positive PRIs to negative PRIs. For each dataset, 10-cross validation is performed on SVM, RF and NB classifiers respectively, and the performance metrics of *SE*, *SP*, *GM* and *ACC* as well as *AUC* are used.

In the sequel, for the simplicity of notation, we denote the ratio of positive samples to negative samples as *PNR*, and remove the words “reliable” and “random” from the dataset names in Table 1. For example, both *SO*_*reliable*_1:1_ and *SO*_*random*_1:1_ are simplified to *SO*_1:1_. In other words, *SO*_1:1_ represents both *SO*_*reliable*_1:1_ and *SO*_*random*_1:1_.

### Performance comparison

**Fig. 4:**
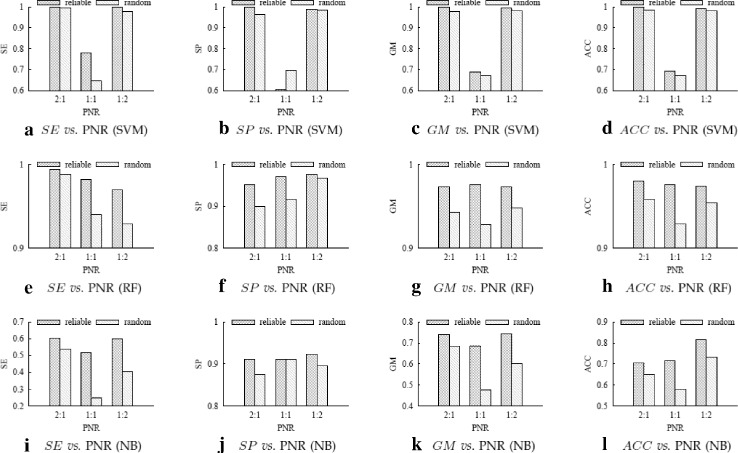
Experimental results on SO datasets. **a**–**d** are the *SE*, *SP*, *GM* and *ACC* values of SVM classifiers; (**e**)–(**h**) are the *SE*, *SP*, *GM* and *ACC* values of RF classifiers; and (**i**)–(**l**) are the *SE*, *SP*, *GM* and *ACC* values of NB classifiers

**Fig. 5:**
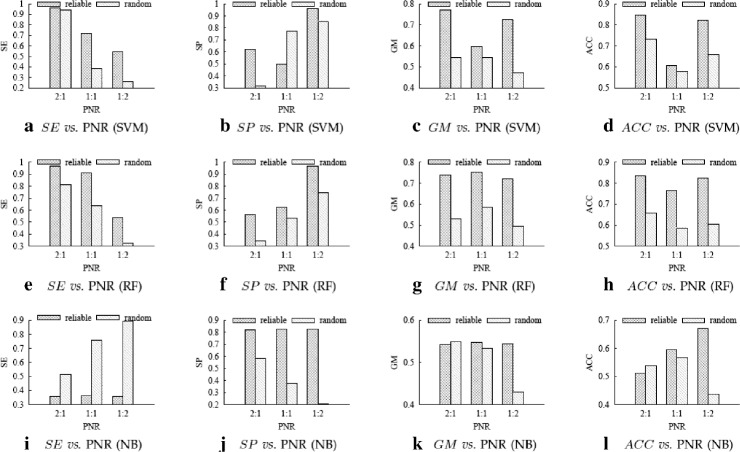
Experimental results on HOMO datasets. **a**–**d** are the *SE*, *SP*, *GM* and *ACC* values of SVM classifiers; (**e**)–(**h**) are the *SE*, *SP*, *GM* and *ACC* values of RF classifiers; and (**i**)–(**l**) are the *SE*, *SP*, *GM* and *ACC* values of NB classifiers

**Fig. 6:**
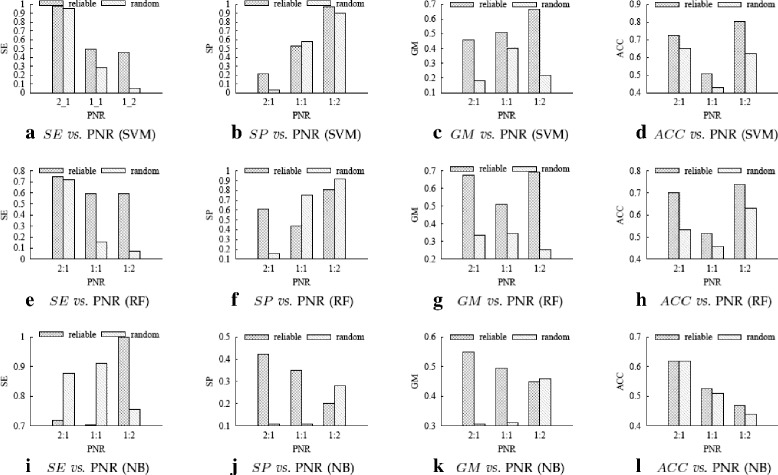
Experimental results on MUS datasets. **a**–**d** are the *SE*, *SP*, *GM* and *ACC* values of SVM classifiers; (**e**)–(**h**) are the *SE*, *SP*, *GM* and *ACC* values of RF classifiers; and (**i**)–(**l**) are the *SE*, *SP*, *GM* and *ACC* values of NB classifiers

Figures 4, 5 and 6 respectively show the performance comparison between using our reliable negative samples and using random negative samples on the *SO* datasets, *HOMO* datasets and *MUS* datasets.

To more clearly evaluate the advantage of reliable negative samples over random negative samples, we define the performance *improvement ratio* (*IR*) of using our reliable negatives over using random negatives as follows:

13 $$ IR = \frac{result_{reliable} - result_{randm}}{result_{random}}\times 100\%,  $$

where *result*_*reliable*_ and *result*_*random*_ denote the performance measure (any of SE, SP, GM and ACC) of using our reliable negatives and using random negatives, respectively. A positive IR means using our reliable negatives achieves better performance than using random negatives. Table 3 shows the *IR* values calculated based on the results in Figs. 4, 5 and 6.

**Table 3:** The improvement ratio (IR) values of different classifiers on different datasets

Dataset	SVM				RF				NB			
	*SE*	*SP*	*GM*	*ACC*	*SE*	*SP*	*GM*	*ACC*	*SE*	*SP*	*GM*	*ACC*
*SO*_1:1_	20.2	–12.8	2.4	3.1	4.5	5.8	5.1	5.1	107.2	0	43.9	23.1
*SO*_1:2_	2.5	0.23	1.4	1.0	4.4	1.0	2.7	2.1	48.4	3.0	23.6	11.4
*SO*_2:1_	0.6	3.7	2.1	1.6	0.6	6.0	3.2	2.3	12.1	4.2	8.1	8.5
*HOMO*_1:1_	86.5	–35.4	9.8	5.3	42.1	17.2	29.0	30.8	–52.2	120.4	2.6	5.0
*HOMO*_1:2_	109.2	12.3	53.3	25.2	65.4	29.0	46.1	35.5	–59.9	302.2	26.9	54.3
*HOMO*_2:1_	2.3	94.5	41.1	15.7	19.1	63.1	39.4	26.8	–30.3	40.3	–1.1	-4.6
*MUS*_1:1_	249.5	–63.4	13	68.7	372.2	–18.7	95.9	53.9	–21.1	299.5	77.5	21.8
*MUS*_1:2_	760.4	7.8	204.5	29.3	751.4	–11.5	174.5	16.7	32.6	–28.1	–2.4	6.7
*MUS*_2:1_	2.7	497.8	147.8	11.7	3.8	286.9	100.4	31.9	–18.0	299.1	80.9	0

From Table 3, we can see that out of the 108 IR values, only 14 IRs are negative, one is 0, the other 93 (93/108 ≈86%) values are positive. As *GM* and *ACC* are more comprehensive than *SE* and *SP* in measuring classification performance, we check their IR values more carefully. Of the 54 IR values for *GE* and *ACC*, 51 (51/54 ≈94%) values are positive. Therefore, in most cases performance measure of our method is better than the random method. The largest IR is 760.4%, which is achieved for *SE* by SVM on dataset *MUS*_1:2_. We can also see that SVM and RF perform better than NB on these datasets.

The results above show that using the reliable negative samples selected by our method indeed boosts the performance of PRI prediction, and our method can serve as a practical and effective method for computationally predicting PRIs.

### The effect of score threshold

To select negative samples, we have to set a score threshold, and require that all candidate negative samples (protein-RNA pairs) have scores (defined in Eq. (1)) no larger than the threshold. So the value of threshold will impact the quality of selected negative samples, and will subsequently impact the prediction performance. The smaller the threshold, the higher the quality of selected negatives, and the smaller the number of negatives that can be selected. So there is a tradeoff between the quality and the number of selected negatives. In this part, we check the impact of score threshold on prediction performance and thus suggest proper values for the threshold. Here, we use *AUC* to evaluate prediction performance.

**Fig. 7:**
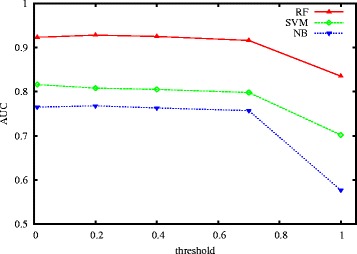
AUC vs. score threshold (RF, SVM and NB)

We randomly select 908 nonredundant positive PRIs of *Homo sapiens* from PRIDB and NPInter, then construct an equal number of negative samples by our method with different score threshold values. Concretely, we generate negative samples like this: give a threshold value *st* (*st* is set to 0, 0.2, 0.4, 0.7 and 1.0 respectively), we select 908 protein-RNA pairs whose scores are closest to *st*. Thus, we construct five PRI datasets. Finally, we evaluate the AUC values of three classifiers RF, SVM and NB on the five constructed datasets by 10-fold cross validation. Figure 7 shows the results. As we can see, for all the three classifiers, with the increase of *threshold* value, the AUC value shows a decreasing trend, which conforms to our expectation. And when the score threshold is less than 0.7, the prediction performance is stable.

### Capability of finding new positive PRIs

In this paper, we define a score (Eq. (1)) to measure the relationship between each protein and each RNA. The smaller the score, the more possible this protein-RNA pair is a negative PRI. Otherwise, the more possible it is a PRI. So the merits of our method are two-fold. On the one hand, we can use it to select highly credible negative PRIs; On the other hand, it can be used to directly predict positive PRIs.

We randomly select 908 nonredundant positive PRIs of *Homo sapiens* from PRIDB and NPInter, and compute the score of any protein-RNA pair not included in the positive set by our method. Among the screened protein-RNA pairs, for each RNA we extract the top 4 protein-RNA pairs in terms of the aggregated score *AS* defined in Eq. (1) and requiring *AS*>1, then we get 397 protein-RNA pairs involving 107 unique RNAs and 96 unique proteins. We search each protein-RNA pair against the NPInter and PRIDB datasets, and find that 22 pairs have been validated by biological experiments.

Furthermore, from the 397 protein-RNA pairs gotten above, we filter out those pairs whose proteins appear in PRIs of the NPInter and PRIDB datasets, and get 256 protein-RNA pairs involving 56 unique RNAs and 74 unique proteins. Then we annotate manually the 74 proteins in the 256 protein-RNA pairs by the Gene Ontology database, and we find that 64 (64/74 ≈86.5%) proteins have RNA binding, chromatin binding or nucleotide binding functions, which play important roles in positive or negative regulation of transcription, gene expression and RNA processing.

**Fig. 8:**
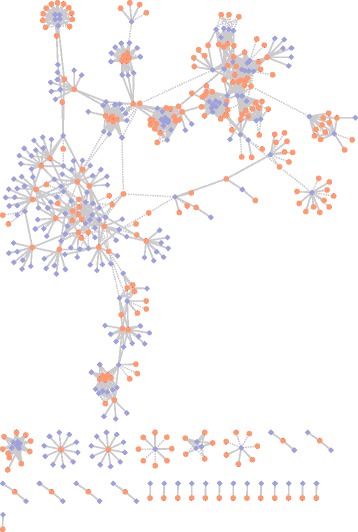
The PRI network constructed by true PRIs and predicted ones by our method. 256 predicted PRIs consist of 74 unique proteins and 56 unique RNAs. The *yellow ellipses* and *purple diamonds* represent proteins and RNAs, respectively. The *solid* and *dotted lines* are the true and predicted PRIs

Figure 8 is a protein-RNA interaction network constructed by the true positive PRIs and the predicted ones. The network includes 908 true PRIs represented by solid line and 256 highly credible predicted PRIs represented by dotted line. Based on our experimental results, we can believe that these predicted PRIs are very possibly true PRIs.

## Conclusion

In this paper, we present a novel method *FIRE* for boosting the performance of protein-RNA interaction prediction by selecting high-quality negative protein-RNA pairs to construct high-performance classifiers. Experiments over 18 PRI datasets show that the three compared classifiers, including SVM, RF and NB all achieve better performance on the negative sets selected by our method than on the random negative sets. This means that our method can screen highly-credible negative PRIs, and thus can improve PRI prediction performance. As for future work, we will further explore the interacting mechanism between protein and RNA, and propose new and more effective methods to select reliable negative samples.
